# Percutaneous left stellate ganglion block for drug-refractory supraventricular arrhythmias in patients with advanced heart failure and cardiogenic shock: a pilot study

**DOI:** 10.1093/europace/euag243

**Published:** 2026-09-03

**Authors:** Filippo Angelini, Arianna Morena, Benedetta De Guidi, Lorenzo Candido Todesco, Pierre Meynet, Carol Gravinese, Daniele Melis, Guglielmo Gallone, Giulia De Lio, Pier Paolo Bocchino, Federico Ferraris, Paolo Boretto, Enrico Baldi, Simone Savastano, Antonio Toscano, Andrea Saglietto, Davide Castagno, Matteo Anselmino, Simone Frea, Gaetano Maria De Ferrari, Veronica Dusi

**Affiliations:** Division of Cardiology, Cardiovascular and Thoracic Department, Città Della Salute e Della Scienza, Corso Bramante 88, 10126, Turin, Italy; Division of Cardiology, Fondazione IRCCS Policlinico San Matteo, Pavia, Italy; RESTART (Cardiac Arrest and Resuscitation Science Research Team), Fondazione IRCCS Policlinico San Matteo, Pavia, Italy; Department of Molecular Medicine, University of Pavia, Pavia, Italy; Department of Medical Sciences, University of Turin, Turin, Italy; Department of Medical Sciences, University of Turin, Turin, Italy; Department of Medical Sciences, University of Turin, Turin, Italy; Division of Cardiology, Cardiovascular and Thoracic Department, Città Della Salute e Della Scienza, Corso Bramante 88, 10126, Turin, Italy; Division of Cardiology, Cardiovascular and Thoracic Department, Città Della Salute e Della Scienza, Corso Bramante 88, 10126, Turin, Italy; Division of Cardiology, Cardiovascular and Thoracic Department, Città Della Salute e Della Scienza, Corso Bramante 88, 10126, Turin, Italy; Department of Medical Sciences, University of Turin, Turin, Italy; Division of Cardiology, Cardiovascular and Thoracic Department, Città Della Salute e Della Scienza, Corso Bramante 88, 10126, Turin, Italy; Division of Cardiology, Cardiovascular and Thoracic Department, Città Della Salute e Della Scienza, Corso Bramante 88, 10126, Turin, Italy; Division of Cardiology, Cardiovascular and Thoracic Department, Città Della Salute e Della Scienza, Corso Bramante 88, 10126, Turin, Italy; Division of Cardiology, Cardiovascular and Thoracic Department, Città Della Salute e Della Scienza, Corso Bramante 88, 10126, Turin, Italy; Division of Cardiology, Fondazione IRCCS Policlinico San Matteo, Pavia, Italy; RESTART (Cardiac Arrest and Resuscitation Science Research Team), Fondazione IRCCS Policlinico San Matteo, Pavia, Italy; Division of Cardiology, Fondazione IRCCS Policlinico San Matteo, Pavia, Italy; RESTART (Cardiac Arrest and Resuscitation Science Research Team), Fondazione IRCCS Policlinico San Matteo, Pavia, Italy; Department of Anaesthesia and Intensive Care Unit-ASL tO4, Ivrea Hospital, Ivrea, Italy; Division of Cardiology, Cardiovascular and Thoracic Department, Città Della Salute e Della Scienza, Corso Bramante 88, 10126, Turin, Italy; Division of Cardiology, Cardiovascular and Thoracic Department, Città Della Salute e Della Scienza, Corso Bramante 88, 10126, Turin, Italy; Department of Medical Sciences, University of Turin, Turin, Italy; Division of Cardiology, Cardiovascular and Thoracic Department, Città Della Salute e Della Scienza, Corso Bramante 88, 10126, Turin, Italy; Department of Medical Sciences, University of Turin, Turin, Italy; Division of Cardiology, Cardiovascular and Thoracic Department, Città Della Salute e Della Scienza, Corso Bramante 88, 10126, Turin, Italy; Division of Cardiology, Cardiovascular and Thoracic Department, Città Della Salute e Della Scienza, Corso Bramante 88, 10126, Turin, Italy; Department of Medical Sciences, University of Turin, Turin, Italy; Division of Cardiology, Cardiovascular and Thoracic Department, Città Della Salute e Della Scienza, Corso Bramante 88, 10126, Turin, Italy; Department of Medical Sciences, University of Turin, Turin, Italy

**Keywords:** Supraventricular arrhythmias, Atrial fibrillation, Acute heart failure, Neuromodulation, Stellate ganglion block

## Background

Managing acute supraventricular arrhythmias (SVAs) in patients with advanced heart failure (HF) and cardiogenic shock is a major therapeutic challenge because conventional rate- and rhythm-control therapies are often limited by hypotension and negative inotropic effects.^1,2^ Percutaneous left stellate ganglion block (PLSGB) is established for drug-refractory ventricular arrhythmias^3,4^ but has never been systematically evaluated for SVAs. Experimental and clinical data suggest that left cardiac sympathetic blockade may reduce both atrial arrhythmia susceptibility^5,6^ and atrioventricular nodal conduction.^7^ We therefore evaluated the feasibility, safety and efficacy of PLSGB for drug-refractory SVAs in critically ill HF patients as a pilot hypothesis-generating study.

## Methods

This single-centre observational study (IRB 0048500) prospectively enrolled consecutive coronary Intensive Care Unit (ICU) patients at Città della Salute e della Scienza, Turin (January 2021–April 2026). Patients received PLSGB for SVAs with a mean heart rate (HR) ≥ 100 bpm and impending/overt haemodynamic deterioration (SCAI shock class ≥ B),^8^ despite optimized medical therapy (intravenous amiodarone ± tolerated beta-blockers). SVAs included paroxysmal AF/atrial tachycardia (PAF/AT), persistent/permanent AF, or atrial flutter (AFL). An ultrasound-guided lateral approach with 5 mL 2% lidocaine and 10 mL 1% ropivacaine was used.^9^

The primary effectiveness endpoint was procedural success at 1 h, defined as either (i) sinus rhythm restoration or complete SVA suppression, or (ii), in patients remaining in SVA, ≥20% HR reduction, without urgent electrical cardioversion or catheter ablation. The principal physiological outcome was HR change from baseline to 1 h. Exploratory analyses included overall and individual components of the primary endpoint, haemodynamic changes at 1 h, SCAI class, vasoactive-inotropic score (VIS), echocardiographic changes at 6 h, and in-hospital outcomes. Procedure-related complications within 24 h were recorded.

## Statistical analysis

Continuous variables are reported as median (IQR) and categorical as *n* (%). Comparisons used non-parametric tests. Analyses were exploratory with unadjusted *P* values; *P* < 0.05 was significant. Analyses were performed in Python at the procedure level, except for patient-level in-hospital outcomes.

## Results

**Figure 1:**
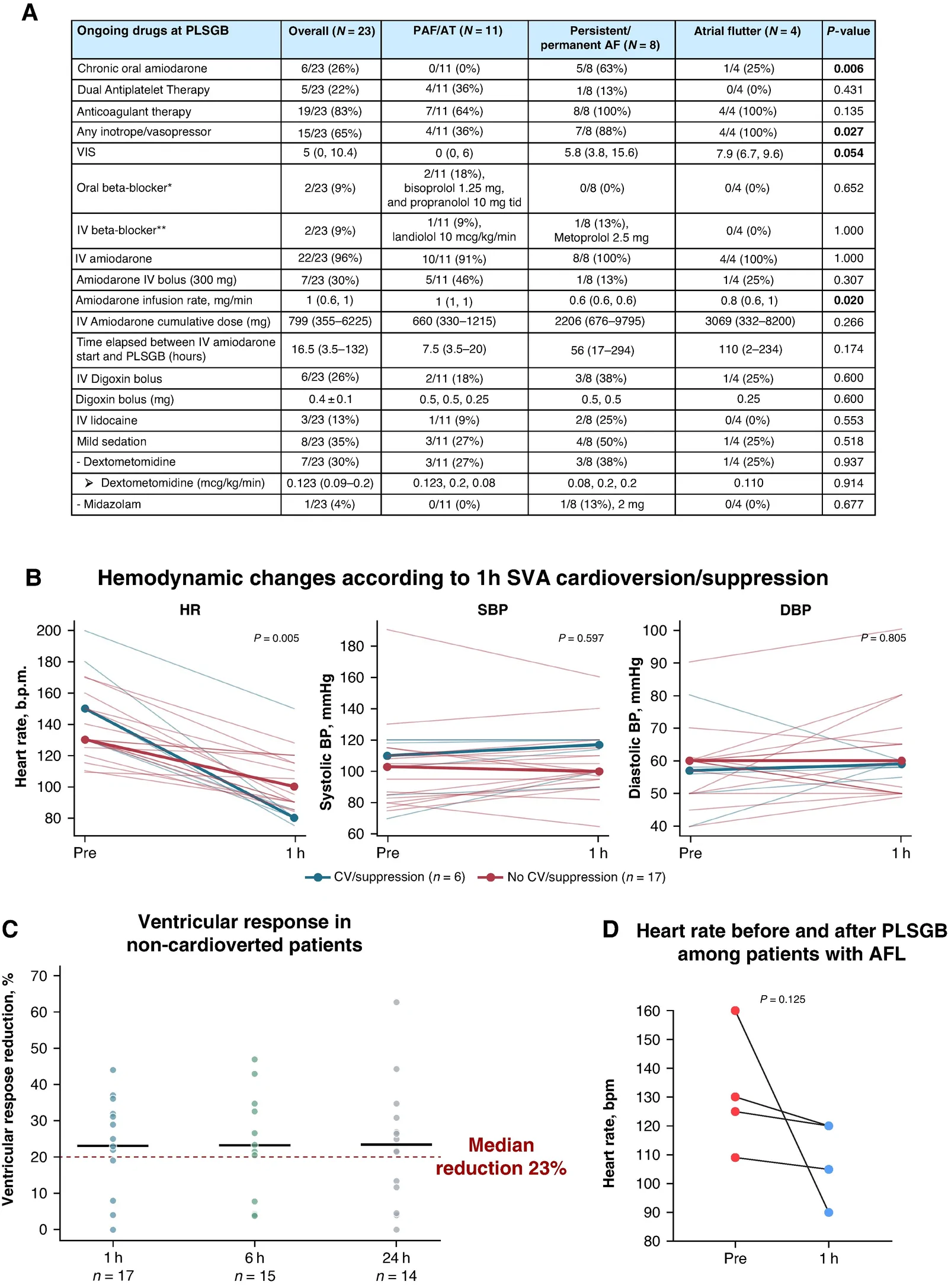
(*A*) Table of ongoing medications at the time of PLSGB across patient groups (overall, PAF/AT, persistent/permanent AF, and atrial flutter), with *P*-values for between-group comparisons. (*B*) Haemodynamic changes from baseline to 1-hour post-PLSGB, stratified by acute SVA cardioversion/suppression. Heart rate decreased in both cardioverted and non-cardioverted patients, with a significantly larger reduction in the former (*P* = 0.005). Systolic (*P* = 0.597) and diastolic BP (*P* = 0.805) remained unchanged in both groups (overall and comparing cardioverted and not cardioverted patients). (*C*) Ventricular response reduction (%) in non-cardioverted patients at 1, 6, and 24 h, with a median reduction of 23% (dashed red line at 20% threshold). (*D*) Heart rate before and 1 h after PLSGB in patients with atrial flutter, showing no significant change (*P* = 0.125). The patient with the highest heart rate was the only one to exhibit a pronounced HR decrease (160 to 90 bpm). Abbreviations: AF, atrial fibrillation; bpm, beats per minute; AT, atrial tachycardia; DAPT, dual antiplatelet therapy; PAF/AT, new onset/paroxysmal AF and atrial tachycardia; SVA, supraventricular arrhythmia; SV, supraventricular; VIS, vasoactive-inotropic score.

Twenty-three PLSGB procedures were performed in 22 patients (median 70 years, 95% male). Indications were PAF/AT (48%), persistent/permanent AF (35%) and AFL (17%, 1 isthmus-dependent). Patients were critically ill, with a median left ventricular ejection fraction 25%, LVOT-VTI 12 cm, left atrial volume index (LAVI) 53 mL/m^2^, NT-proBNP 6664 ng/L, creatinine 1.5 mg/dL, and nearly half in SCAI class C/D. Approximately one-third had at least moderate mitral regurgitation, and over half had right ventricle dysfunction. ICU admissions were mostly due to acute HF (61%) and acute coronary syndrome (26%). Non-ischaemic cardiomyopathy was present in 39%, one third had an ICD. Half had prior SVA, 17% with prior ablation. The majority (65%) required vasoactive therapy, 22% mechanical circulatory support and 17% non-invasive ventilation. Ongoing medications (*Figure 1A*) included IV beta-blockers (9%), IV amiodarone (96%, median infusion rate1 mg/min), IV digoxin (25%), and IV sedation (35%). Median systolic/diastolic blood pressure (SBP/DBP) was 103/60 mmHg. Median HR was 130 bpm (*P* = 0.025 across groups, 150 bpm in PAF/AT vs. 130 in persistent/permanent AF and 128 in AFL).

Overall procedural success was 78% (18/23 procedures, 95% CI 58–90%), lower in AFL (25%, *P* = 0.041), with cardioversion or complete SVA suppression occurring in 26% of patients (6/23), driven exclusively by the PAF/AT group (55% vs. 0% in the other 2 groups, *P* = 0.015). A HR reduction ≥20% occurred in 71% (12/17) of the non-cardioverted patients and accounted for most treatment responses (12/18, 67%). Procedural success remained 70% and 74% at 6 and 24 h. respectively. Complete SVA suppression within 24 h occurred in 35%, exclusively in PAF/AT (73% vs. 0%, *P* < 0.001). Urgent electrical cardioversion was needed in 13% by 24 h. Smaller LAVI was associated with 1-hour cardioversion (42 vs. 57 mL/m^2^, *P* = 0.039).

One-hour post-PLSGB, HR decreased from 130 (IQR 129–150) to 90 bpm (IQR 90–115) (*P* < 0.001), with a greater reduction in cardioverted patients (Δ −60 vs. −35 bpm, *P* = 0.005), while BP remained stable (*Figure 1B*). Non-cardioverted patients maintained a median 23% HR reduction for up to 24 h (*Figure 1C*). None of the four AFL patients cardioverted; only one showed a significant, sustained HR reduction at 1 h lasting 24 h (*Figure 1D*).

By 6 h, median LVOT-VTI improved from 12 to 13 cm (*P* = 0.004, median paired increase 22%) and amiodarone infusion rate was reduced from 1.0 to 0.6 mg/min (*P* = 0.020), with IV amiodarone use decreasing from 96% to 87%, and no changes in the VIS score.

No major complications occurred; minor and self-resolving complications (17%) included hoarseness (13%), brachial plexus anaesthesia (13%), and dysphagia (4%). Median length of stay was 20 days. Ineffective PLSGB showed higher, albeit not significant, need for urgent cardioversion/ablation (33% vs. 6%, *P* = 0.169). Four patients died (18%), three received LVAD, one underwent heart transplant.

## Discussion

This pilot study provides the first systematic clinical experience with one shot PLSGB for drug-refractory SVAs in critically ill patients with advanced HF. Four observations emerge.

First, the predominant effect appears to be rapid ventricular rate control without compromising blood pressure, consistent with preferential modulation of AV nodal sympathetic input.

Second, rhythm conversion occurred almost exclusively in paroxysmal atrial arrhythmias, suggesting that atrial sympathetic modulation is more effective before advanced atrial structural remodelling develops.

Third, the negative dromotropic effect of PLSGB seems less pronounced in AFL, likely reflecting the inability of a partial sympathetic modulation to shift a stable, organized 2:1 or 3:1 block into a higher degree of block unless the baseline sympathetic drive is extraordinarily high.

Lastly, no major procedure-related complications occurred. PLSGB reduced amiodarone requirements, supporting its role as an adjunct—not an alternative—to conventional antiarrhythmic therapy.

Limitations include its single-centre nature with no control group, modest sample size, and concomitant amiodarone use. The additional benefit of continuous PLSGB after 6 h remains to be established.^10^

## Conclusions

One-shot PLSGB provided rapid and haemodynamically neutral ventricular rate control in critically ill HF patients with drug-refractory SVAs while facilitating rhythm conversion in selected patients with paroxysmal atrial arrhythmias. These preliminary findings support further prospective evaluation of PLSGB as an adjunctive neuromodulator strategy in acute HF and cardiogenic shock.

## Data Availability

The data underlying this article will be shared on reasonable request to the corresponding author.
